# Differences in blood levels of neuroligin-derived peptides in a cohort for early detection of Alzheimer’s disease

**DOI:** 10.1093/gerona/glag009

**Published:** 2026-02-03

**Authors:** Milton G F Fernandes, Maxime Pinard, Esen Sokullu, Cyntia Tremblay, Jean-François Gagnon, Frédéric Calon, Benoit Coulombe, Jonathan Brouillette

**Affiliations:** Department of Pharmacology and Physiology, Faculty of Medicine, Université de Montréal, Montreal, Québec, Canada; Translational Proteomics Laboratory, Institut de Recherches Cliniques de Montréal, Montreal, Québec, Canada; Translational Proteomics Laboratory, Institut de Recherches Cliniques de Montréal, Montreal, Québec, Canada; Neuroscience Axis CHU de Québec Research Center, Laval University, Quebec City, Québec, Canada; Department of Psychology, Université du Québec à Montréal, Montreal, Québec, Canada; Centre for Advanced Research in Sleep Medicine, Hôpital du Sacré-Coeur de Montréal, Centre intégré universitaire de santé et de services sociaux du Nord de l’Île-de-Montréal, Montreal, Québec, Canada; Neuroscience Axis CHU de Québec Research Center, Laval University, Quebec City, Québec, Canada; Faculty of Pharmacy, Laval University, Quebec City, Québec, Canada; Translational Proteomics Laboratory, Institut de Recherches Cliniques de Montréal, Montreal, Québec, Canada; Department of Pharmacology and Physiology, Faculty of Medicine, Université de Montréal, Montreal, Québec, Canada

**Keywords:** CIMA-Q, Neurodegeneration, Neuroligin, Synapse, Tau

## Abstract

Alzheimer’s disease (AD) develops gradually, with significant neurodegeneration already present by the time clinical symptoms emerge. Since synapses are affected early in the disease, synaptic proteins are being investigated as potential markers of the prodromal stage. Using data and plasma samples provided by the Consortium for the early identification of Alzheimer’s disease—Quebec, we analyzed plasma levels of neuroligin (NLGN)-derived peptides in cognitively normal (CN) individuals and cognitively impaired (CI) individuals, including those with amnestic mild cognitive impairment and early-stage AD. Plasma levels of NLGN-derived peptides were assessed by quantifying tryptic peptides using liquid chromatography coupled with tandem mass spectrometry. Our findings show that levels of specific NLGN peptides were significantly elevated in CI compared to CN individuals. Receiver operating characteristic curve analysis revealed that some NLGN peptides could distinguish CI individuals. Furthermore, analysis based on mini-mental state examination scores revealed that specific plasma phosphorylated tau peptides were significantly and positively correlated with selected NLGN-derived peptides in more advanced stages of cognitive decline. These results support further investigation into synaptic NLGN-derived peptides in the blood as promising tools for monitoring the earliest stages of AD.

## Graphical abstract

**Graphical Abstract:**
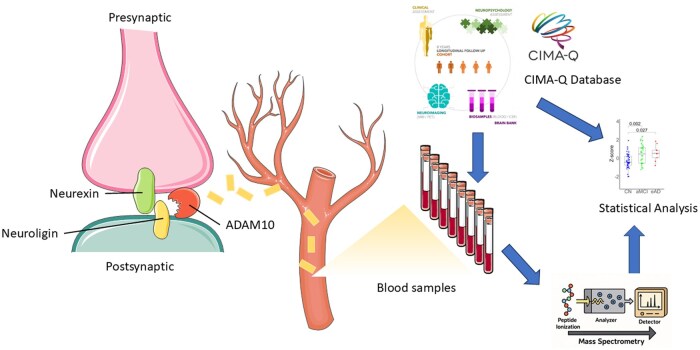

## Introduction

Alzheimer’s disease (AD) is the most common cause of dementia and represents a major public health challenge worldwide. Early detection is crucial for improving patient outcomes and for the timely implementation of therapeutic strategies. Before the full clinical expression of AD dementia, amnestic mild cognitive impairment (aMCI) has been identified as a key transitional stage,^1^^,^^2^ that offers a critical prodromal window for diagnosis, prevention, and treatment. However, quantitative tools to detect the underlying neuropathology in aMCI are still limited at present.

The Consortium for the early identification of Alzheimer’s disease—Quebec (CIMA-Q) cohort established in 2013 includes clinically, cognitively, and neuroimaging-characterized elderly men and women from whom blood samples were collected. The goals of this cohort are: to enable early diagnosis of AD; to provide a well-characterized dataset for the scientific community; to identify new therapeutic targets to prevent or slow cognitive decline in AD; and to support subsequent clinical studies.^3^ As such, the CIMA-Q cohort offers a convenient database for the study of tools for early detection of aMCI and early AD (eAD).

Synapse loss is a hallmark of prodromal AD and a major predictor of cognitive decline.^4^^,^^5^ While it is well established that synaptic degeneration is driven by neuronal hyperactivity rather than hypoactivity particularly at the onset of the disease,^6^^,^^7^ the specific proteins affected by this glutamatergic hyperactivity remain poorly characterized. Given that synaptic dysfunction and loss precede neuronal death, identifying proteins altered at synapses during the prodromal stage of AD could enable earlier detection—prior to the onset of irreversible neurodegeneration and severe brain damage.

Neuroligin-1 (NLGN1) is a postsynaptic adhesion protein localized at excitatory synapses, where it interacts with the presynaptic protein neurexin and plays a critical role in synapse formation, maintenance, and plasticity.^8-10^ Although all neuroligin isoforms (NLGN1, NLGN2, NLGN3, NLGN4X, and NLGN4Y) are enriched in postsynaptic densities, NLGN1 is the only 1 predominantly localized at excitatory synapses.^11^ Previous studies have shown that neuronal hyperactivity promotes cleavage of the extracellular domain of NLGN1,^12^^,^^13^ which is subsequently released into the blood.^14^^,^^15^

Recently, our laboratory found that individuals with aMCI exhibit lower post mortem levels of NLGN1 in the hippocampus, using an antibody targeting its extracellular domain.^16^ Moreover, NLGN1 levels were further reduced in AD patients compared to both aMCI individuals and age-matched nondemented controls.^16^ We also observed that lower hippocampal levels of NLGN1 were associated with higher hippocampal levels of soluble amyloid-beta oligomers (Aβo), and poorer cognitive performance, as measured by the mini-mental state examination (MMSE) in individuals with aMCI and AD.^16^ These findings suggest that measuring NLGN1 levels targeting the extracellular region could help distinguish individuals with aMCI from patients with AD or cognitively healthy elderly, and thus support the monitoring of disease progression.

Synaptosomal degeneration has also been observed in GABAergic inhibitory interneurons in certain brain regions of patients with AD and animal models.^17^ Given that neuroligin-2 (NLGN2) is exclusively localized at inhibitory synapses,^18^ we also aimed to assess whether this isoform is altered in the plasma of individuals with aMCI and early AD.

Thus, we hypothesize that the neuronal hyperactivity observed in prodromal AD induces NLGN cleavage, leading to a progressive increase in soluble NLGN-derived peptides in the plasma of individuals with aMCI and early AD compared to age-matched, cognitively normal (CN) elderly.

In this study, we investigated whether plasma levels of NLGN-derived peptides are altered in individuals with cognitive impairment (CI), including those with aMCI and early AD, compared to CN participants coming from the CIMA-Q cohort, by measuring tryptic peptides using liquid chromatography coupled to tandem mass spectrometry (LC-MS/MS). We also examined the correlation between NLGN peptide levels and plasma concentrations of phosphorylated Tau peptides (pTau), which have recently emerged as promising predictors of AD.^19^ Additionally, we assessed whether NLGN peptide levels are associated with cognitive performance, hippocampal atrophy, and other variables (age, sex, and APOE4 genotype) linked to aMCI and early AD. Finally, we examined whether measuring plasma NLGN peptide levels can help distinguish CI from CN individuals.

## Methods

### Participants

The data used in this manuscript were obtained in part from the Consortium for the early identification of CIMA-Q, established in 2013 with initial funding from FRQS-Pfizer. The designated principal investigator and director of CIMA-Q is Dr Sylvie Belleville from the Research Center of the Institute Universitaire de Gériatrie de Montréal (IUGM), a research organization within the Integrated University Health and Social Services Centre of the Centre-South of Montreal Island. CIMA-Q represents the collaborative efforts of many co-principal investigators and researchers from Quebec affiliated with Université Laval, McGill University, Université de Montréal, and Université de Sherbrooke. Since 2014, CIMA-Q has recruited over 420 participants aged 65 and older who are CN, have MCI or AD.

The CIMA-Q is a multicenter longitudinal, parallel group study, recruiting volunteers from memory clinics, media advertisements and the NuAge population study in the province of Quebec, Canada. Participants pass clinical evaluations, psychometric testing and have blood sample collected, optionally consenting for MRI, PET, and CSF collection. Inclusion and exclusion criteria are summarized in **Table S1**. Further details on recruitment methods, exclusion and inclusion criteria, testing materials, and general methodology for the CIMA‐Q project can be found in Belleville et al.^3^

Diagnostic criteria for AD and aMCI were based on the National Institute on Aging—Alzheimer’s Association and were described in detail in Belleville et al.^3^ The diagnostic classification of CIMA-Q participants is provided in **Table S2**, while the demographic characteristics of the plasma samples used in this study are provided in **Table S3**.

### PAC-qMS sample preparation for NLGN levels measurement

Five micrograms of specific antibody against both NLGN1 (Bio-techne, AF4340) and NLGN2 (Bio-techne, AF5645) was coupled overnight at 37 °C to 1 mg of dynabead epoxy M-270 (Thermofisher scientific, Cat #: 14311D) and equilibrated according to the manufacturer’s specifications. Samples and calibration points were prepared by using similar amounts of either patient plasma or commercially availed pooled plasma (Innovative research, Cat #: IPLANAC50ML), respectively. Each sample was diluted in a final volume of 250 µL with 1x PBS (Wisent, Cat #: 311-010-EL). Calibration curves were generated by spiking diluted pooled plasma with known amounts of the recombinant NLGN1 protein (Cedarlane, Cat #: TP307524) and NLGN2 protein (Bio-techne, Cat #: 5645-NL_050) (0, 1, 2, 5, 10, 25, and 100 ng). A calibration curve was prepared for each set of 34 tested patient plasma. Equilibrated antibody-coupled magnetic beads were added to the plasma samples (patient or calibration curve points) and NLGN1-NLGN2 proteins capture was performed at 4 °C for 1 h on a rotator shaker. Magnetic beads were washed 6 times with fresh HBSEP buffer (10 mM Tris-HCl pH 8.0, 150 mM NaCl, 3 mM EDTA, 0.005% Tween20) and 4 times with water. Captured proteins were eluted with a 33% acetonitrile and 0.4% trifluoroacetic acid elution buffer, dried down by using a speed vacuum centrifuge (Eppendorf) and stored at −70 °C until sample digestion. The dried pellets were resuspended in 20 µL of 6 M urea and 100 mM ammonium bicarbonate. Five microliters of reduction buffer (45 mM dithiothreitol, 100 mM ammonium bicarbonate) was added to each sample and incubated at 37 °C for 30 min on a thermomixer (Thermofisher scientific) set at 350 RPM. Five microliters of alkylation buffer was added to each tube (100 mM iodoacetamide, 100 mM ammonium bicarbonate) and protein alkylation was performed at 24 °C for 20 min in the dark. Prior to trypsin digestion, 50 µL of 50 mM ammonium bicarbonate was added to reduce the urea concentration under 2 M. Trypsin solution, 5 µL of 100 ng/µL of trypsin sequencing grade from Promega (Promega, Cat #: V5111), 50 mM ammonium bicarbonate was added to each sample. Protein digestion was performed at 37 °C for 18 h. The protein digests were acidified with 9.4 µL formic acid 5% in water and dried with a speed vacuum centrifuge. Prior to LC-MS/MS, protein digests were re-solubilized under agitation for 15 min in 12 µL of 2% ACN-1% formic acid and spiked with heavy-labeled standard peptides specific to NLGN1 and NLGN2 protein forms (JPT Peptide Technologies Gmbh). These peptides were added for signal normalization and retention time confirmation of 1 NLGN1 peptide (GNYGLLDLIQAL-R*) and 1 NLGN2 peptide (FPVVNTAYG-R*) where R* is Arg U-^13^C6 and U-^15^N4. Desalting and cleanup of the digests were performed using C18 ZipTip pipette tips (Millipore). Eluates were dried down in speed vacuum centrifuge and stored at −20°C until LC-MS/MS analysis.

### Mass spectrometry of NLGN peptides

Samples were reconstituted under agitation for 15 min in 11 µL of 2% ACN-1% FA and loaded into a 75 μm i.d. × 150 mm Self-Pack C18 column installed in the Easy-nLC II system (Proxeon Biosystems). The buffers used for chromatography were 0.2% formic acid (buffer A) and ACN/0.2% formic acid (buffer B). Peptides were eluted with a 2-slope gradient at a flowrate of 250 nL/min. Solvent B first increased from 2% to 35% for 40 min and then from 35% to 85% for 5 min. The HPLC system was coupled to an Orbitrap Fusion mass spectrometer (Thermo Scientific) through a Nanospray Flex Ion Source. Nanospray and S-lens voltages were set to 1.3-1.8 kV and 60 V, respectively. The capillary temperature was set to 250 °C. Targeted MS^2^ spectra were acquired in the Orbitrap with a resolution of 30 000, a target value at 5e4 and a maximum injection time of 150 ms. The peptide fragmentation was performed in the HCD cell at a normalized collision energy of 29%. The peptide *m/z* and retention time used to detect each peptide are listed in the **Table S4**.

### Plasma levels of phosphorylated tau measurements

Plasma levels of pTau181 and pTau231 were measured using Simoa technology as previously described.^20^^,^^21^ Measurement of plasma levels of pTau217 was executed using a S-PLEX Human Tau kit for pTau217 detection (MSD, K151APFS).

### Data analysis

Raw mass spectrometry data were imported in PinPoint (ThermoFisher scientific, Version 1.4.0). Tryptic peptide abundance of each peptide with at least 2 major ion transitions similar to those detected in the calibration curve were considered for further analysis and each participant sample was calculated using the sum of the area of the ion transitions per peptides and normalized against the sum of the area of 4 transition of the heavy-labeled NLGN1 peptide (**Figure S1**). Injected amount (in ng) was used to determine the standard curve linearity coefficient for each tryptic peptide and to calculate the linear regression equation (**Figure S2**). NLGNs tryptic peptide amount (in ng) for each sample was evaluated by using the regression equation and the concentration in each sample was then calculated. Lower limit of detection (LLOD) and lower limit of quantification (LLOQ) were set as 3 and 10 times the background level measured in the lowest amount of the calibration curve.

### Statistical analysis

All statistical analyses were executed in R.

#### Sample size and statistical power

For ANCOVA analyses, statistical power was estimated using the pwr.anova.test() function from the pwr R package, assuming α = 0.05 and a medium effect size (Cohen’s *f* = 0.30) adjusted for covariates according to:


$$f_{\mathrm{adj}}=f\times \sqrt{\frac{1}{1-{R^{2}}_{\mathrm{covariates}}}},$$

where $R_{\text{covariates}}^{2}$ represents the proportion of variance in the dependent variable explained by covariates (age, sex, and APOE genotype).

The effective sample size (*n*_eff_) for different group sizes was calculated as:


(1) $$n_{\mathrm{eff}}=\frac{k}{\sum_{1}^{k}\left(\frac{1}{n_{i}}\right)},$$

where *k* is the number of groups and *n_i_* the number of participants per group.

For partial correlation analyses, the statistical power controlled for covariates was estimated using the pwr.f2.test function from the *pwr* package in R, assuming a medium effect size (partial *r* = 0.30) and a significant level of 0.05.

The statistical power for detecting a significant difference between the area under the receiver operating characteristic (ROC) curve (AUC) and the null value of 0.5 was estimated using the power.roc.test() function from the *pROC* R package, assuming an expected AUC of 0.75 and α = 0.05.

The estimated statistical power for comparisons between CN vs CI, aMCI or eAD groups are presented in **Tables S5 and S6**.

#### Normalization and batch effect correction

After the logarithm transformation (base 2) of the concentration of each NLGN tryptic peptide, values were normalized to eliminate the batch effect, according to the following equation:


(2) $$Z_{i,j}=\frac{\left(C_{i,j}-X_{j}\right)}{\sigma_{j}}$$

where:

*Z_i,j_* = *Z*-score of the sample *i* of the batch *j*

*C_i,j_* = Log_2_ of the tryptic peptide concentration of the sample *i* of the batch *j*

*X_j_* = Mean of the log_2_ of the tryptic peptide concentration of the batch *j*

*σ_j_* = Standard deviation of the log_2_ of the tryptic peptide concentration of the batch *j*

#### Statistical tests

##### ANCOVA and Kruskal–Wallis analyses

The Shapiro–Wilk test and the Levene’s test were used to check for normality and homoscedasticity. ANCOVA, including age, sex, and APOE genotype as covariates, or the Kruskal–Wallis test, were performed to assess the differences in groups analyses, and the Tukey’s test, or the Dunn’s test, for differences between pairs, in cases with multiple groups. *P*-values were adjusted for false discovery rate (FDR).

##### Correlation analyses

Multiple linear regression models accounting for covariates (age, sex, and APOE genotype) were used to calculate β coefficients and *p*-values. Partial Pearson correlation coefficients were calculated assuming age, sex, and APOE genotype as covariates. *P*-value were adjusted for FDR.

##### ROC curves and AUC with cross-validation analysis

ROC analyses were performed using logistic regression models including relevant covariates (age, sex, and APOE genotype). Ten-fold cross-validation repeated 5 times was used to assess model performance. Out-of-fold predicted probabilities were pooled to obtain a cross-validated ROC curve and corresponding AUC with 95% confidence intervals (DeLong method).

## Results

### Plasma levels of NLGN2(41-50) and NLGN2(336-346) were elevated in CI individuals

**Figure 1.**
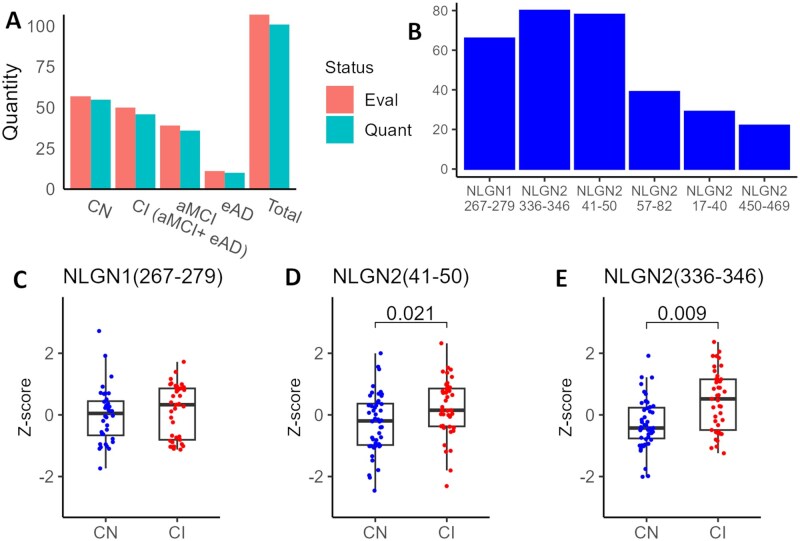
Plasma levels of NLGN-associated peptides in CI individuals—combining those with aMCI and eAD—compared to CN individuals from the CIMA-Q cohort. (A) Number of samples evaluated and successfully quantified by diagnostic group. (B) Number of samples in which each peptide was quantified. (C-E) Plasma levels of NLGN-derived peptides in CN and CI groups. *Z*-score corresponds to the normalized value corrected for batch effect. Statistical differences were assessed using ANCOVA, including age, sex and APOE genotype as covariates (NLGN2[336-346] and NLGN2[41-50]) and the Kruskal–Wallis test (NLGN1[267-279]). *P*-values were adjusted for false discovery rate (FDR). aMCI, amnestic mild cognitive impairment; APOE, apoliprotein E; CI, cognitively impaired; CIMA-Q, Consortium for the early identification of Alzheimer's disease-Quebec; CN, cognitively normal; eAD, early Alzheimer’s disease; NLGN, neuroligin.

A total of 107 plasma samples from the CIMA-Q dataset were evaluated, and the levels of 6 NLGN-associated peptides were successfully quantified in 101 of them. The distribution of samples by diagnosis group (CN, aMCI, eAD, and CI—grouping aMCI and eAD) is shown in Figure 1A. Figure 1B presents the number of samples in which the 5 NLGN2 peptides and the single NLGN1 peptide were quantified. The amino acids sequences of these peptides are listed in **Table S4**, and their positions within the NLGN1 and NLGN2 proteins are shown in **Figure S3**. Peptides NLGN1(267-279), NLGN2(336-346), and NLGN2(41-50) were detected in most samples (Figure 1B). NLGN2(17-40), NLGN2(57-82), and NLGN2(450-469) were detected in only a few, and due to the low quantity of samples they were not further analyzed.

Given that aMCI is often considered a prodromal stage of AD, we grouped aMCI and eAD individuals under the broader category of cognitively impaired (CI) individuals to assess peptide performance across the early stages of the disease continuum. The levels of NLGN2(41-50) and NLGN2(336-346) were significantly higher in CI compared to CN individuals (Figure 1D and E). No significant differences were observed in NLGN1(267-279) levels (Figure 1C). However, a significant difference in NLGN1(267-279) levels was detected in participants with eAD compared to CN individuals, although the statistical power was limited due to the small sample size of the eAD group (**Figure S4A**).

We also evaluated the correlation between known AD risk factors (APOE genotype, sex, and age) and plasma levels of NLGN-derived peptides. No significant differences were found according to these factors, although a trend was observable for APOE4 carriers and with increasing age (**Figure S5**).

### Correlation analyses between plasma levels of NLGN-derived peptides, plasma levels of phosphorylated tau peptides at residues 181, 217, and 231, and hippocampal volume

**Figure 2.**
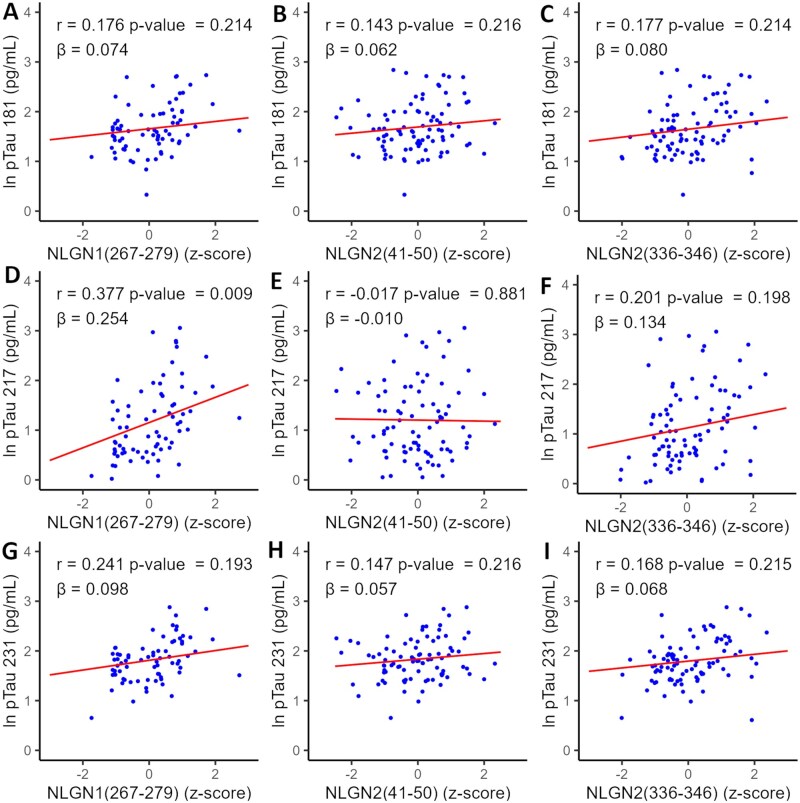
Plasma levels of NLGN-derived peptides in relation to phosphorylated tau (A-I) correlations between plasma concentration of NLGN1(267-279), NLGN2(41-50), and NLGN2(336-346) and pTau 181, 217, and 231. Correlations were derived from linear models including age, sex, and APOE genotype as covariates. Pearson partial correlation coefficients, significance levels, and beta coefficients are shown at the top of each plot. NLGN peptide levels are expressed as *Z*-scores, normalized and corrected for batch effects. P-values were adjusted for FDR. APOE, apoliprotein E; NLGN, neuroligin; pTau, phosphorylated tau.

Plasma levels of pTau have been identified as a promising indicator of AD.^19^^,^^22^ We therefore evaluated the correlation between plasma concentration of pTau and NLGN-derived peptides. A particularly meaningful correlation is observed between NLGN1(267-279) and pTau 217 (Figure 2D). No significant correlations were found between the remaining NLGN peptides and any of the pTau species, although an overall trend for positive correlation can be observed (Figure 2).

Another well-known feature of AD is the reduction in hippocampal volume.^23^ Taking advantage of the availability of this measurement in the CIMA-Q cohort, we assessed the correlation between plasma NLGN peptide levels and hippocampal volume. Although this measure was available for only a subset of samples, reducing the statistical power of the analysis, an inverse correlation can be observed between peptides and the hippocampal volume, though the associations did not reach conventional statistical significance (*p*-value = .06 for NLGN2) (**Figure S6**).

### Associations between plasma levels of NLGN-associated peptides, MMSE scores, and pTau plasma levels

To further explore the association between NLGN peptides and pTau across dementia stages, we stratified participants into 3 groups based on MMSE scores: normal (>25), mild (20-24), and moderate (10-19). Notably, no patients were classified as severe (MMSE < 10), as the CIMA-Q cohort primarily includes cognitively healthy individuals or those in the early stages of dementia, with most falling into the normal or mild categories. Given the limited number of participants in the moderate group, we combined mild and moderate cases for analysis.

**Figure 3.**
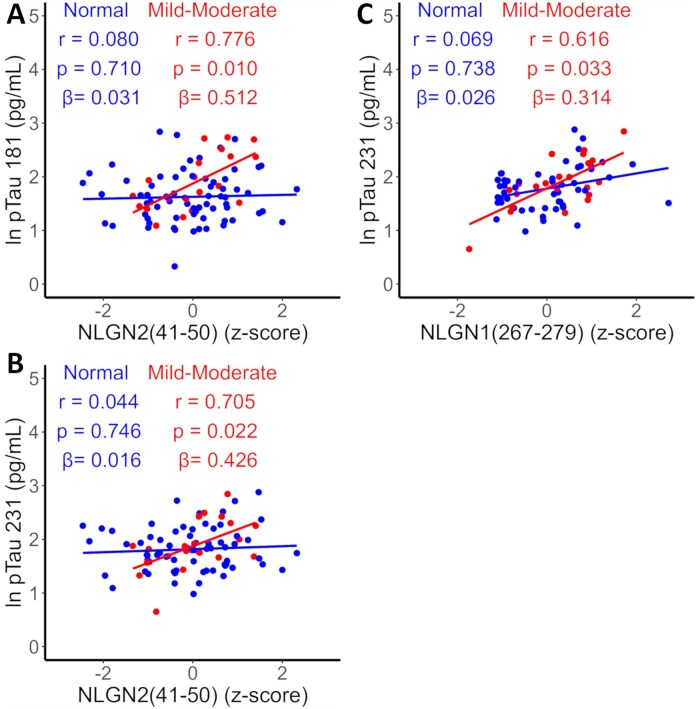
Plasma levels of NLGN-derived peptides in relation to mini-mental state examination (MMSE) score and pTau. (A-C) Correlations between plasma levels of NLGN2(41-50) and NLGN1(267-279), and plasma levels of phosphorylated tau at residues 181 and 231, stratified by cognitive severity based on MMSE score. Participants were grouped into 2 categories: normal (MMSE 30-25), and mild-moderate (MMSE 24-10). correlations were derived from linear models including age, sex and APOE genotype as covariates. Pearson partial correlation coefficients, significance levels, and beta coefficients are shown at the top of each plot. NLGN peptide levels are expressed as *Z*-scores, normalized and corrected for batch effects. P-values were adjusted for false discovery rate (FDR). APOE, apoliprotein E; NLGN, neuroligin; pTau, phosphorylated tau.

We observed an increased correlation between plasma levels of some NLGN-associated peptides and pTau concentrations in individuals with mild-moderate dementia compared to those classified as normal (Figure 3). This increase was significant for the following pairs: NLGN2 (41-50) and pTau 181 (Figure 3A); NLGN2(41-50) and pTau 231(Figure 3B); NLGN1(267-279) and pTau 231 (Figure 3C). No significant increase was found in the other pairs (**Figure S7**).

### Potential of NLGN-associated peptides plasma levels to distinguish patients with aMCI

**Figure 4.**
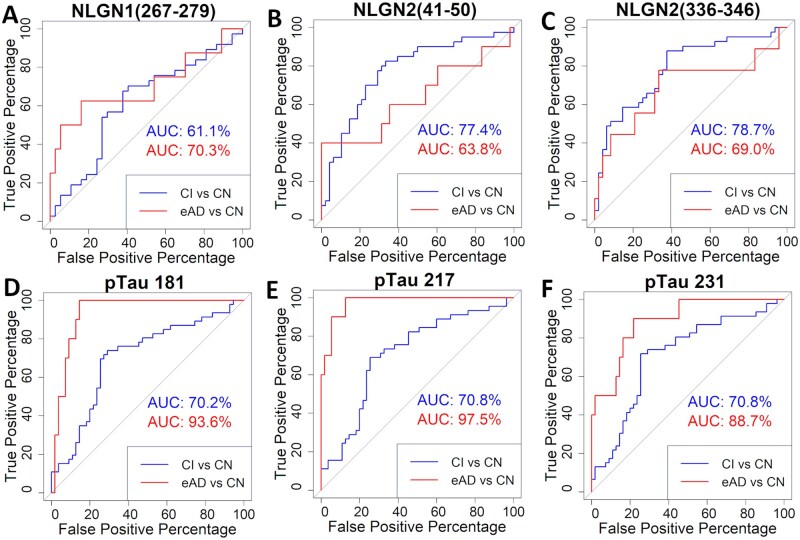
Potential of plasma levels of NLGN-derived peptides to distinguish CIs and those with eAD. (A-F) Receiver operating characteristic (ROC) curves with area under the curve (AUC) values for NLGN peptides (A-C), and phosphorylated tau (D-F) comparing CI vs CN (blue line), and eAD only vs CN (red line). ROC analyses were performed using logistic regression models including age, sex, and APOE genotype as covariates. Model performance was evaluated using 10-fold cross-validation repeated 5 times. CI, cognitively impaired; CN, cognitively normal; eAD, early Alzheimer’s disease; NLGN, neuroligin; pTau, phosphorylated tau.

We further evaluated the potential of the plasma NLGN-derived peptide levels to distinguish between CN, CI, and eAD cases. To this end, we analyzed receiver operating characteristic (ROC) curves and calculated the AUC for all peptides, comparing individuals with cognitive impairment (CI—aMCI and eAD combined) to the CN group, as well as cases of eAD alone to CN (Figure 4). This analysis took into account age, sex, and APOE genotype as confounding variables.

For CI versus CN, we observed AUC values above 75% for NLGN2(41-50) (Figure 4B) and NLGN2(336-346) (Figure 4C) and above 60% for NLGN1(267-270) (Figure 4A). For eAD versus CN, AUC values exceeded 70% for NLGN1(267-279) (Figure 4A).

Plasma levels of pTau have been proposed as a strong tool to distinguish cases of AD.^19^ Accordingly, we evaluated the ROC curves and AUC for plasma concentration of pTau at 3 epitopes (pTau 181, pTau 217, and pTau 231), and compared them to those obtained for NLGN1/2 peptides. Notably, all 3 pTau showed AUC values above 85% when comparing the CN and eAD groups, whereas the AUC values for CI vs CN were around 70% (Figure 4D-F).

## Discussion

The findings of this study reveal that specific NLGN-derived peptides are elevated in CI individuals compared to CN participants from the CIMA-Q cohort. Plasma NLGN1(267-279) levels showed significant correlation with pTau 217 concentrations, and the correlations between NLGN1(267-279) and NLGN2(41-50) with pTau 181 and 231 were stronger in individuals with mild-to-moderate cognitive impairment, as defined by the MMSE score. Additionally, measuring plasma NLGN peptide levels may aid in distinguishing CI individuals, particularly when NLGN2 and phosphorylated tau (pTau) are assessed in combination. While NLGN2 better discriminated CI individuals, pTau was more indicative of eAD.

A previous study using targeted mass spectrometry reported increased levels of NLGN2-derived peptides—specifically NLGN2(336-346), which were also analyzed in our study—in the cerebrospinal fluid (CSF) of patients with preclinical AD.^24^ However, their approach failed to detect in CSF the NLGN1 peptide they used, which differed from the 1 used in our analysis. Another study found that CSF levels of NLGN1 peptide were significantly reduced in patient with mild to moderate AD compared to controls, although there was substantial overlap between groups.^25^ In contrast, no significant differences in NLGN1 or NLGN2 peptide levels were observed in the CSF of patients with MCI and AD in a study using parallel reaction monitoring mass spectrometry.^26^ Notably, the three NLGN1 peptides assessed in that study differed from the one used in our study, and only one of the two NLGN2 peptides we examined in greater depth was included. These methodological differences likely account, at least in part, for the discrepancies observed across studies.

Given the conflicting results in CSF and the potential differences between profiles in CSF and plasma, our study specifically investigated NLGN1 and NLGN2 peptide levels in the plasma of individuals with aMCI and AD. To this end, we developed an ultra-sensitive mass spectrometry approach capable of simultaneously analyzing the levels of the extracellular NLGN1 and NLGN2 peptides by targeted LC-MS/MS analysis of specific tryptic peptides within the same NLGN1/2-enriched plasma sample.

NLGN1 is cleaved by the same enzymes involved in the processing of amyloid precursor protein (APP) to generate the soluble Aβ peptides. Indeed, the metalloproteinase ADAM10 cleaves the extracellular domain of NLGN1, while the γ-secretase processes the remaining membrane-bound fragment by cleaving its intracellular domain.^12^ Additionally, activity-induced NLGN1 cleavage has been shown to be influenced by the matrix metalloproteinase MMP9,^13^ an enzyme involved in many processes implicated in the development of AD.^27^ Our results support the notion that neuronal hyperactivity in the prodromal stage of AD promotes the cleavage of the NLGN extracellular domain, leading to a progressive decrease in soluble NLGN peptides in the brain, as we previously reported,^16^ and a corresponding increase in their release into the blood. These findings align with a previous study showing reduced levels of NLGN in neuron-derived plasma exosomes from patients with AD, using an antibody targeting the extracellular domain of NLGN.^28^ A key advantage of analyzing NLGN peptides directly in plasma, as done in our study, is that it offers a simpler, faster, and more cost-effective approach compared to isolating exosomes.

Changes in plasma levels of pTau and Aβ peptides support the amyloid cascade hypothesis, which posits the accumulation of Aβ and pTau in the brain are central hallmarks of AD.^29^ This hypothesis provides logical rationale for using these molecules as indicators of the disease. However, incorporating additional pathological features of AD could strengthen diagnostic approaches by diversifying detection strategies. One such feature is synaptic degeneration, a well-recognized process in AD pathogenesis.^30^ Notably, synaptic loss occurs early in the disease course,^31^^,^^32^ offering a valuable opportunity for the development of early-stage detection methods. Several synaptic proteins have been proposed as CSF-based indicators of AD, particularly the postsynaptic protein neurogranin and the presynaptic proteins synaptosomal-associated protein 25 (SNAP25) and synaptotagmin-1 (SYT1).^33-35^ While neurogranin has shown promise as a CSF-based indicator of AD, its plasma levels appear unchanged, likely due to substantial extracerebral production that may mask brain-derived alterations.^36^^,^^37^ In contrast, recent studies suggest that β-synuclein and SNAP25 can be detected in plasma and may serve as potential indicators of AD-related synaptic changes.^38-40^ However, further research is required to validate these findings.

We did not identify a strong correlation between NLGN-peptides levels and age, although a trend was observed. However, our sample included only individuals aged 65 years and older, with a mean age of 74, representing a relatively restricted age range. Studies including participants across a broader age spectrum would help clarify the influence of age on the release of these peptides into the bloodstream. Furthermore, investigations into the mechanisms underlying the transport of synaptic proteins-derived peptides from the brain to the blood—particularly those affected by aging or by pathologies characterized by synaptic degenerations, potentially involving the glymphatic system^41^—will be important to better understand this process in the context of AD.

In our study, we evaluate the potential of NLGN-derived peptides as indicators of synapse dysfunction to distinguish early stages of AD. Although the sample size was smaller than in other cohorts, a major strength of this work lies in its focus on the prodromal and early clinical stages of the disease—enabled by the unique design of the CIMA-Q cohort, which was specifically established to support the early detection of AD.

This study has some limitations that should be addressed in future research to confirm our findings and further evaluate the potential of plasma NLGN peptides to distinguish aMCI and early AD. Although our sample size of 101 participants was sufficient to detect significant differences in plasma levels of various tryptic peptides derived from digested, enriched NLGNs between CI and CN groups, it remains relatively small compared with larger cohorts commonly used to evaluate disease-related indicators. A larger sample size would help determine whether differences exist between aMCI and eAD groups, as only a few samples were available for the latter due to the particular design of the CIMA-Q. This limitation reflects a trade-off with the unique design of the CIMA-Q cohort, which is specifically focused on early detection of AD. This focus also constrains the strength of the correlation between NLGN-derived peptides and hippocampus volume, since hippocampal atrophy is more prominent in later stages of the disease.

Moreover, the correlation between plasma NLGN2 peptide levels and pTau may be modest because these two features are associated with distinct disease stages, as suggested by our MMSE-based stratification (Figure 3). Adding participants with later-stage AD would also allow a clearer assessment of this relationship. Although the CIMA-Q cohort is well characterized clinically, cognitively, and through neuroimaging, it lacks plasma measurements of Aβ_42_ and Aβ_40_, and no PET imaging data for Aβ and Tau are currently available. In addition, only a small number of participants underwent lumbar puncture for CSF analysis of Aβ and Tau levels. Studying NLGN plasma levels in other cohorts will further clarify how these peptides relate to other hallmarks of AD.^42-45^

Although NLGN1 and NLGN2 are predominantly expressed in the brain, they are also detected in peripheral tissues according to the Human Protein Atlas. However, the significant differences observed between CI and CN individuals suggest that peripheral expression of NLGN1 and NLGN2 is relatively stable in our samples and does not mask CNS-related differences between groups. Incorporating paired CSF and PET imaging data in future studies could help confirm that the observed alterations in NLGN peptides originate from the CNS.

## Conclusions

In recent years, considerable attention has been given to blood-based markers such as pTau and Aβ_42/40_ ratio as predictors of AD.^46^^,^^47^ However, less emphasis has been placed on identifying indicators that reflect fundamental pathological processes like synapse loss, one of the strongest predictors of cognitive decline in AD.^4^^,^^5^ To our knowledge, this is the first study to report elevated plasma levels of NLGN2-derived peptides in individuals with aMCI and early AD. The findings of this study suggest that plasma levels of NLGN-derived peptides may reflect synaptic dysfunction associated with AD, and could help identify the disease at an early stage.

## Supplementary material

Supplementary data are available at *The Journals of Gerontology, Series A: Biological Sciences and Medical Sciences* online.

## Supplementary Material

glag009_Supplementary_Data

## Data Availability

The data that support the findings of this study are available from the corresponding author upon reasonable request.

## References

[1] Anderson ND. State of the science on mild cognitive impairment (MCI). CNS Spectr. 2019;24:78-87. 10.1017/s1092852918001347 30651152

[2] Vos SJ , van Rossum IA, Verhey F, et al Prediction of Alzheimer disease in subjects with amnestic and nonamnestic MCI. Neurology. 2013;80:1124-1132. 10.1212/WNL.0b013e318288690c 23446677

[3] Belleville S , LeBlanc AC, Kergoat M-J, et al The consortium for the early identification of Alzheimer’s disease–Quebec (CIMA-Q). Alz Dem Diag Ass Dis Mo. 2019;11:787-796. 10.1016/j.dadm.2019.07.003 10.1016/j.dadm.2019.07.003 PMC6880140 31788534

[4] Selkoe DJ. Alzheimer’s disease is a synaptic failure. Science. 2002;298:789-791. 10.1126/science.1074069 12399581

[5] Coleman PD , Yao PJ. Synaptic slaughter in Alzheimer’s disease. Neurobiol Aging. 2003;24:1023-1027. 10.1016/j.neurobiolaging.2003.09.001 14643374

[6] Busche MA , Chen X, Henning HA, et al Critical role of soluble amyloid-beta for early hippocampal hyperactivity in a mouse model of Alzheimer’s disease. Proc Natl Acad Sci USA. 2012;109:8740-8745. 10.1073/pnas.1206171109 22592800 PMC3365221

[7] Busche MA , Grienberger C, Keskin AD, et al Decreased amyloid-beta and increased neuronal hyperactivity by immunotherapy in Alzheimer’s models. Nat Neurosci. 2015;18:1725-1727. 10.1038/nn.4163 26551546

[8] Südhof TC. Neuroligins and neurexins link synaptic function to cognitive disease. Nature. 2008;455:903-911. 10.1038/nature07456 18923512 PMC2673233

[9] Craig AM , Kang Y. Neurexin-neuroligin signaling in synapse development. Curr Opin Neurobiol. 2007;17:43-52. 10.1016/j.conb.2007.01.011 17275284 PMC2820508

[10] Lee AK , Khaled H, Chofflet N, Takahashi H. Synaptic organizers in Alzheimer’s disease: a classification based on amyloid-beta sensitivity. Front Cell Neurosci. 2020;14:281. 10.3389/fncel.2020.00281 32982693 PMC7492772

[11] Song JY , Ichtchenko K, Sudhof TC, Brose N. Neuroligin 1 is a postsynaptic cell-adhesion molecule of excitatory synapses. Proc Natl Acad Sci USA. 1999;96:1100-1105. 10.1073/pnas.96.3.1100 9927700 PMC15357

[12] Suzuki K , Hayashi Y, Nakahara S, et al Activity-dependent proteolytic cleavage of neuroligin-1. Neuron. 2012;76:410-422. 10.1016/j.neuron.2012.10.003 23083742

[13] Peixoto RT , Kunz PA, Kwon H, et al Transsynaptic signaling by activity-dependent cleavage of neuroligin-1. Neuron. 2012;76:396-409. 10.1016/j.neuron.2012.07.006 23083741 PMC3783515

[14] Keshishian H , Burgess MW, Gillette MA, et al Multiplexed, quantitative workflow for sensitive biomarker discovery in plasma yields novel candidates for early myocardial injury. Mol Cell Proteomics. 2015;14:2375-2393. 10.1074/mcp.M114.046813 25724909 PMC4563722

[15] Keshishian H , Burgess MW, Specht H, et al Quantitative, multiplexed workflow for deep analysis of human blood plasma and biomarker discovery by mass spectrometry. Nat Protoc. 2017;12:1683-1701. 10.1038/nprot.2017.054 28749931 PMC6057147

[16] Dufort-Gervais J , Provost C, Charbonneau L, et al Neuroligin-1 is altered in the hippocampus of Alzheimer’s disease patients and mouse models, and modulates the toxicity of amyloid-beta oligomers. Sci Rep. 2020;10:6956. 10.1038/s41598-020-63255-6 32332783 PMC7181681

[17] Garcia-Marin V , Blazquez-Llorca L, Rodriguez JR, et al Diminished perisomatic GABAergic terminals on cortical neurons adjacent to amyloid plaques. Front Neuroanat. 2009;3:28. 10.3389/neuro.05.028.2009 19949482 PMC2784678

[18] Ali H , Marth L, Krueger-Burg D. Neuroligin-2 as a central organizer of inhibitory synapses in health and disease. Sci Signal. 2020;13:1-13. 10.1126/scisignal.abd8379 33443230

[19] Barthélemy NR , Salvadó G, Schindler SE, et al Highly accurate blood test for Alzheimer’s disease is similar or superior to clinical cerebrospinal fluid tests. Nat Med. 2024;30:1085-1095. 10.1038/s41591-024-02869-z 38382645 PMC11031399

[20] Karikari TK , Pascoal TA, Ashton NJ, et al Blood phosphorylated tau 181 as a biomarker for Alzheimer’s disease: a diagnostic performance and prediction modelling study using data from four prospective cohorts. Lancet Neurol. 2020;19:422-433. 10.1016/s1474-4422(20)30071-5 32333900

[21] Ashton NJ , Pascoal TA, Karikari TK, et al Plasma p-tau231: a new biomarker for incipient Alzheimer’s disease pathology. Acta Neuropathol. 141:709-724. 10.1007/s00401-021-02275-6 PMC8043944 33585983

[22] Barthélemy NR , Horie K, Sato C, Bateman RJ. Blood plasma phosphorylated-tau isoforms track CNS change in Alzheimer’s disease. J Exp Med. 2020;217:1–11. 10.1084/jem.20200861 32725127 PMC7596823

[23] Balestrieri JVL , Nonato MB, Gheler L, Prandini MN. Structural volume of hippocampus and Alzheimer’s disease. Rev Assoc Med Bras. 2020;66:512-515. 10.1590/1806-9282.66.4.512 32578788

[24] Lleó A , Núñez-Llaves R, Alcolea D, et al Changes in synaptic proteins precede neurodegeneration markers in preclinical Alzheimer’s disease cerebrospinal fluid. Mol Cell Proteomics. 2019;18:546-560. 10.1074/mcp.RA118.001290 30606734 PMC6398205

[25] Camporesi E , Lashley T, Gobom J, et al Neuroligin-1 in brain and CSF of neurodegenerative disorders: investigation for synaptic biomarkers. Acta Neuropathol Commun. 2021;9:19. 10.1186/s40478-021-01119-4 33522967 PMC7852195

[26] Camporesi E , Nilsson J, Vrillon A, et al Quantification of the trans-synaptic partners neurexin-neuroligin in CSF of neurodegenerative diseases by parallel reaction monitoring mass spectrometry. EBioMedicine. 2022;75:103793. 10.1016/j.ebiom.2021.103793 34990894 10.1016/j.ebiom.2021.103793 PMC8743209

[27] Rosenberg GA. Matrix metalloproteinases and their multiple roles in neurodegenerative diseases. Lancet Neurol. 2009;8:205-216. 10.1016/s1474-4422(09)70016-x 19161911

[28] Goetzl EJ , Abner EL, Jicha GA, Kapogiannis D, Schwartz JB. Declining levels of functionally specialized synaptic proteins in plasma neuronal exosomes with progression of Alzheimer’s disease. Faseb J. 2018;32:888-893. 10.1096/fj.201700731R 29025866 PMC5888398

[29] Kepp KP , Robakis NK, Høilund-Carlsen PF, Sensi SL, Vissel B. The amyloid cascade hypothesis: an updated critical review. Brain. 2023;146:3969-3990. 10.1093/brain/awad159 37183523

[30] Tzioras M , McGeachan RI, Durrant CS, Spires-Jones TL. Synaptic degeneration in Alzheimer disease. Nat Rev Neurol. 2023;19:19-38. 10.1038/s41582-022-00749-z 36513730

[31] Masliah E , Mallory M, Alford M, et al Altered expression of synaptic proteins occurs early during progression of Alzheimer’s disease. Neurology. 2001;56:127-129. 10.1212/wnl.56.1.127 11148253

[32] Scheff SW , Price DA, Schmitt FA, DeKosky ST, Mufson EJ. Synaptic alterations in CA1 in mild Alzheimer disease and mild cognitive impairment. Neurology. 2007;68:1501-1508. 10.1212/01.wnl.0000260698.46517.8f 17470753

[33] Hellwig K , Kvartsberg H, Portelius E, et al Neurogranin and YKL-40: independent markers of synaptic degeneration and neuroinflammation in Alzheimer’s disease. Alzheimers Res Ther. 2015;7:74. 10.1186/s13195-015-0161-y 26698298 PMC4690296

[34] Kvartsberg H , Portelius E, Andreasson U, et al Characterization of the postsynaptic protein neurogranin in paired cerebrospinal fluid and plasma samples from Alzheimer’s disease patients and healthy controls. Alzheimers Res Ther. 2015;7:40. 10.1186/s13195-015-0124-3 26136856 PMC4487851

[35] Brinkmalm A , Brinkmalm G, Honer WG, et al SNAP-25 is a promising novel cerebrospinal fluid biomarker for synapse degeneration in Alzheimer’s disease. Mol Neurodegener. 2014;9:53. 10.1186/1750-1326-9-53 25418885 PMC4253625

[36] De Vos A , Jacobs D, Struyfs H, et al C-terminal neurogranin is increased in cerebrospinal fluid but unchanged in plasma in Alzheimer’s disease. Alzheimers Dement. 2015;11:1461-1469. 10.1016/j.jalz.2015.05.012 26092348

[37] Arslan B , Zetterberg H, Ashton NJ. Blood-based biomarkers in Alzheimer’s disease—moving towards a new era of diagnostics. Clin Chem Lab Med. 2024;62:1063-1069. 10.1515/cclm-2023-1434 38253262

[38] Oeckl P , Anderl-Straub S, Danek A, et al Relationship of serum beta-synuclein with blood biomarkers and brain atrophy. Alzheimers Dement. 2023;19:1358-1371. 10.1002/alz.12790 36129098

[39] Oeckl P , Janelidze S, Halbgebauer S, et al Higher plasma β-synuclein indicates early synaptic degeneration in Alzheimer’s disease. Alzheimers Dement. 2023;19:5095-5102. 10.1002/alz.13103 37186338

[40] Nilsson J , Ashton NJ, Benedet AL, et al Quantification of SNAP-25 with mass spectrometry and Simoa: a method comparison in Alzheimer’s disease. Alzheimers Res Ther. 2022;14:78. 10.1186/s13195-022-01021-8 35659284 PMC9166380

[41] Rasmussen MK , Mestre H, Nedergaard M. Fluid transport in the brain. Physiol Rev. 2022;102:1025-1151. 10.1152/physrev.00031.2020 33949874 PMC8897154

[42] Olsson B , Lautner R, Andreasson U, et al CSF and blood biomarkers for the diagnosis of Alzheimer’s disease: a systematic review and meta-analysis. Lancet Neurol. 2016;15:673-684. 10.1016/s1474-4422(16)00070-3 27068280

[43] Cohen AD , Landau SM, Snitz BE, Klunk WE, Blennow K, Zetterberg H. Fluid and PET biomarkers for amyloid pathology in Alzheimer’s disease. Mol Cell Neurosci. 2019;97:3-17. 10.1016/j.mcn.2018.12.004 30537535

[44] Ossenkoppele R , van der Kant R, Hansson O. Tau biomarkers in Alzheimer’s disease: towards implementation in clinical practice and trials. Lancet Neurol. 2022;21:726-734. 10.1016/s1474-4422(22)00168-5 35643092

[45] Öhrfelt A , Brinkmalm A, Dumurgier J, et al The pre-synaptic vesicle protein synaptotagmin is a novel biomarker for Alzheimer’s disease. Alzheimers Res Ther. 2016;8:41. 10.1186/s13195-016-0208-8 27716408 PMC5048479

[46] Blennow K , Zetterberg H. Biomarkers for Alzheimer’s disease: current status and prospects for the future. J Intern Med. 2018;284:643-663. 10.1111/joim.12816 30051512

[47] Karikari TK , Ashton NJ, Brinkmalm G, et al Blood phospho-tau in Alzheimer disease: analysis, interpretation, and clinical utility. Nat Rev Neurol. 2022;18:400-418. 10.1038/s41582-022-00665-2 35585226

