# Supplementary material for: Differences in blood levels of neuroligin-derived peptides in a cohort for early detection of Alzheimer’s disease
Source: J Gerontol A Biol Sci Med Sci. 2026 Feb 3;81(4):glag009. doi: 10.1093/gerona/glag009 (PMC13049705; doi:10.1093/gerona/glag009)
Supplement: glag009_Supplementary_Data [file glag009_supplementary_data.zip › Supplementary material JG Review Final.pdf]

SUPPLEMENTARY TABLES & FIGURES

Differences in blood levels of neuroligin-derived peptides in a cohort for early detection of Alzheimer's disease

Milton Guilherme Forestieri Fernandes, PhD[1], Maxime Pinard, PhD[2], Esen Sokullu, PhD[2], Cyntia Tremblay, MS[6], Jean-François Gagnon, PhD[3,4], Frédéric Calon, PhD[5,6], Benoit Coulombe, PhD[2,] the Consortium for the early identification of Alzheimer's disease-Quebec (CIMA-Q)*, Jonathan Brouillette, PhD[1] **

Affiliations:

1 Department of Pharmacology and Physiology, Faculty of Medicine, Université de Montréal, Montreal, QC, Canada, H3T 1J4.

2 Translational Proteomics Laboratory, Institut de Recherches Cliniques de Montréal, Montreal, QC, Canada, H2W 1R7.

3 Department of Psychology, Université du Québec à Montréal, Montreal, QC, Canada, H2X 3P2.

4 Centre for Advanced Research in Sleep Medicine, Hôpital du Sacré-Coeur de Montréal, Centre intégré universitaire de santé et de services sociaux du Nord de l'Île-de-Montréal, Montreal, Canada, H4J 1C5.

5 Faculty of Pharmacy, Laval University, Quebec, QC, Canada, G1V 0A6.

6 Neuroscience Axis CHU de Québec Research Center – Laval University, Quebec, QC, Canada, G1V 4G2.

* The data used in the preparation of this article were obtained thanks to the Consortium for the early identification of Alzheimer's disease – Quebec (CIMA-Q; cima-q.ca). A list of researchers involved in the design of CIMA-Q can be found on the cima-q.ca website. These researchers contributed to the establishment of protocols, the implementation of the research infrastructure, the recruitment and follow-up of participants, the obtaining of data, the maintenance of biological and ex-vivo samples, and certain derived data.

**** Corresponding authors:**

Jonathan Brouillette

phone: 514 338-2222 #3359

email: jonathan.brouillette@umontreal.ca

Supplementary Table 1. Inclusion and exclusion criteria – CIMA-Q

Supplementary Table 2. Test results for each diagnostic group – CIMA-Q

Supplementary Table 3. Participants characteristics – CIMA-Q.

Supplementary Table 4. Peptide sequences and their mass spectrometry characteristics.

Supplementary Table 5. Statical power for ANCOVA and ROC analyses

Supplementary Table 6. Statical power for correlation analyses

Supplementary Figure 1. NLGNs-enrichment assay chromatograms.

Supplementary Figure 2. Standard curves with recombinant NLGNs

Supplementary Figure 3. Position of the peptides measured in NLGN1 and NLGN2 amino acid sequence.

Supplementary Figure 4. Plasma levels of NLGN-associated peptides (A)NLGN1(267-279), (B) NLGN2(41-50), and (C) NLGN2(336-346) in cognitively normal (CN) individuals compared to individuals with amnestic mild cognitive impairment (aMCI) and early Alzheimer's disease (eAD)

Supplementary Figure 5. Blood concentration of NLGN peptides by APOE4 genotype, sex and age.

Supplementary Figure 6. Plasma concentration of NLGN-associated peptides in relation to the size of hippocampus.

Supplementary Figure 7. Plasma levels of NLGN-associated peptides in relation to Mini-Mental State Examination (MMSE) score and pTau.

Supplementary Table 1. Inclusion and exclusion criteria – CIMA-Q

Inclusion Criteria	Exclusion Criteria
1. Being 65 years old and over<br>2. Living in a community or residence for an independent person (or an equivalent living environment)<br>3. Have a score on the telephone-mini mental state examination (T-MMSE) of 17 or higher (≥17 / 26).<br>4. Understand, read and write French or English<br>5. Have sufficient visual and auditory acuity to be able to go through the neuropsychology tests visit.<br>6. For participants with mild Alzheimer's dementia (AD), be accompanied during clinical visits. For other participants, have an informant to answer questions (in person or by phone or in writing).<br>7. Be willing to: answer questionnaires about one's state of health; have a physical and neuropsychological evaluation; submit to a blood test.	1. Plan to move out of Quebec in the next three years<br>2. Have a score of 20 or higher (≥20 / 27) on the Patient Health Questionnaire-9 (PHQ-9) scale.<br>3. Have a score on the Clinical dementia rating (CDR) greater than 1 (> 1).<br>4. Have a disease or impairment of the central nervous system, including:<br>• Subdural hematoma (active or past)<br>• Subarachnoid hemorrhage (active or past)<br>• Primary or metastatic brain cancer<br>• Epilepsy (active)<br>• Dementia (other than mild Alzheimer's disease) or other neurodegenerative diseases<br>5. Have had intracranial surgery.<br>6. Have an active addiction to alcohol, drugs or narcotics.<br>7. Have regular consumption of benzodiazepines greater than the equivalent of 1 mg per day lorazepam taken orally.<br>8. Have an illness / condition that is related to cognitive impairment or one that could interfere with the subject's participation in the project.

Note. Abbreviations: T-MMSE, Telephone-Mini Mental State Examination; AD, Alzheimer’s Disease.

Supplementary Table 2. Test results for each diagnostic group

Test or Questionnaire	CN	aMCI	eAD
**MoCA**	≥ 26 / 30	20-25	13-25
**Wechsler**	I. ≥ 9 for 16 years and more of education<br>II. ≥ 5 for 8-15 years of education<br>III. ≥ 3 for 0-7 years or education	I. ≤ 11 for 16 years and more of education<br>II. ≤ 9 for 8-15 years of education<br>III. ≤ 6 for 0-7 years of education	I. ≤ 8 for 16 years and more of education<br>II. ≤ 4 for 8-15 years of education<br>III. ≤ 2 for 0-7 years of education
**CDR**	0	0.5	(0.5) 1
**NIA-AA**		Fit the clinical criteria of NIA-AA for MCI-AD.	Fit the clinical criteria of NIA-AA for probable Alzheimer.

Note. Abbreviations: CN, Cognitively Normal; MCI, Mild Cognitive Impairment; AD, Alzheimer's Disease; MoCA, Montreal Cognitive Assessment; CDR; Clinical Dementia Rating; NIA-AA, National Institute on Aging and Alzheimer's Association.

Supplementary Table 3. Participants characteristics – CIMA-Q.

	CN (n=55)	aMCI (n=36)	eAD (n=10)	CI (aMCI+eAD) (n=46)
**Age, mean (sd)**	71.8 (5.0)	76.0 (6.4) *	77.1 (6.4) *	76.2 (6.3) *
**Women, n (%)**	40 (72%)	18 (50%) *	6 (60%)	24 (52%)
**APOE4 carrier, n (%)**	11 (20.0%)	10 (27.8%)	6 (60.0%) *	16 (34.7%)
**MMSE, mean score**	24.8	24.0	21.1*	23.3*

Note. Abbreviations: CN – cognitively normal, aMCI – amnestic mild cognitive impairment, eAD – early Alzheimer's disease, CI – cognitively impaired.
* Statistically significantly higher than the cognitively normal group at the 5% significance level.

Supplementary Table 4. Peptide sequences and their mass spectrometry characteristics.

Endogenous peptide	m/z	z	t start (min)	t stop (min)	Peptide sequence
**NLGN1 (267-279)**	**723.4092**	**2**	**47.3**	**56**	**(K)GNYGLLDLIQALR(W)**
**NLGN1 (267-279) - Heavy**	**728.4134**	**2**	**47.3**	**56**	**(K)GNYGLLDLIQALR(W) heavy**
**NLGN2 (17-40)**	**981.9854**	**2**	**32.5**	**38.3**	**(R)GGGGPGGGAPGGPGLGLGSLGEER(F)**
**NLGN2 (41-50)**	**562.2984**	**2**	**29.3**	**34.5**	**(R)FPVVNTAYGR(V)**
**NLGN2 (41-50) - Heavy**	**567.3026**	**2**	**29.3**	**34.5**	**(R)FPVVNTAYGR(V) heavy**
**NLGN2 (57-82)**	**922.5070**	**3**	**50**	**57**	**(R)ELNNEILGPVVQFLGVPYATPPLGAR(R)**
**NLGN2 (336-346)**	**635.3258**	**2**	**24**	**29.3**	**(R)ELVDQDVQPAR(Y)**
**NLGN2 (450-469)**	**718.3970**	**3**	**43**	**50**	**(K)TLLALFTDHQWVAPAVATAK(L)**

Footnote: The position of the starting and ending amino acids of each peptide are indicated in parentheses in the first column.

Supplementary Table 5. Statical power for ANCOVA and ROC analyses

Variable	Groups compared	Control sample size	Case sample size	Power ANCOVA	Power ROC
NLGN1(267-279)	CN, CI	37	37	0.74	0.98
NLGN2(41-50)	CN, CI	48	40	0.81	0.99
NLGN2(336-346)	CN, CI	48	41	0.85	0.99
NLGN1(267-279)	CN, aMCI, eAD	37	29 (aMCI), 8 (eAD)	0.44	-
NLGN2(41-50)	CN, aMCI, eAD	48	30 (aMCI), 10 (eAD)	0.52	-
NLGN2(336-346)	CN, aMCI, eAD	48	32 (aMCI), 9 (eAD)	0.53	-
pTAU 181	CN, CI	55	46	-	0.99
pTAU 217	CN, CI	54	46	-	0.99
pTAU 231	CN, CI	55	46	-	0.99
NLGN1(267-279)	CN, eAD	37	8	-	0.63
NLGN2(41-50)	CN, eAD	48	10	-	0.72
NLGN2(336-346)	CN, eAD	48	9	-	0.69
pTAU 181	CN, eAD	55	10	-	0.74
pTAU 217	CN, eAD	54	10	-	0.74
pTAU 231	CN, eAD	55	10	-	0.74

Supplementary Table 6. Statical power for correlation analyses

Variable 1	Variable 2	Sample size	Power Partial Correlations
NLGN1(267-279)	pTau 181, pTau 217, pTau 231	74	0.74
NLGN2(41-50)	pTau 181, pTau 217, pTau 231	88	0.82
NLGN2(336-346)	pTau 181, pTau 217, pTau 231	89	0.82
NLGN1(267-279)	Hippocampus	37	0.43
NLGN2(41-50)	Hippocampus	44	0.50
NLGN2(336-346)	Hippocampus	44	0.50

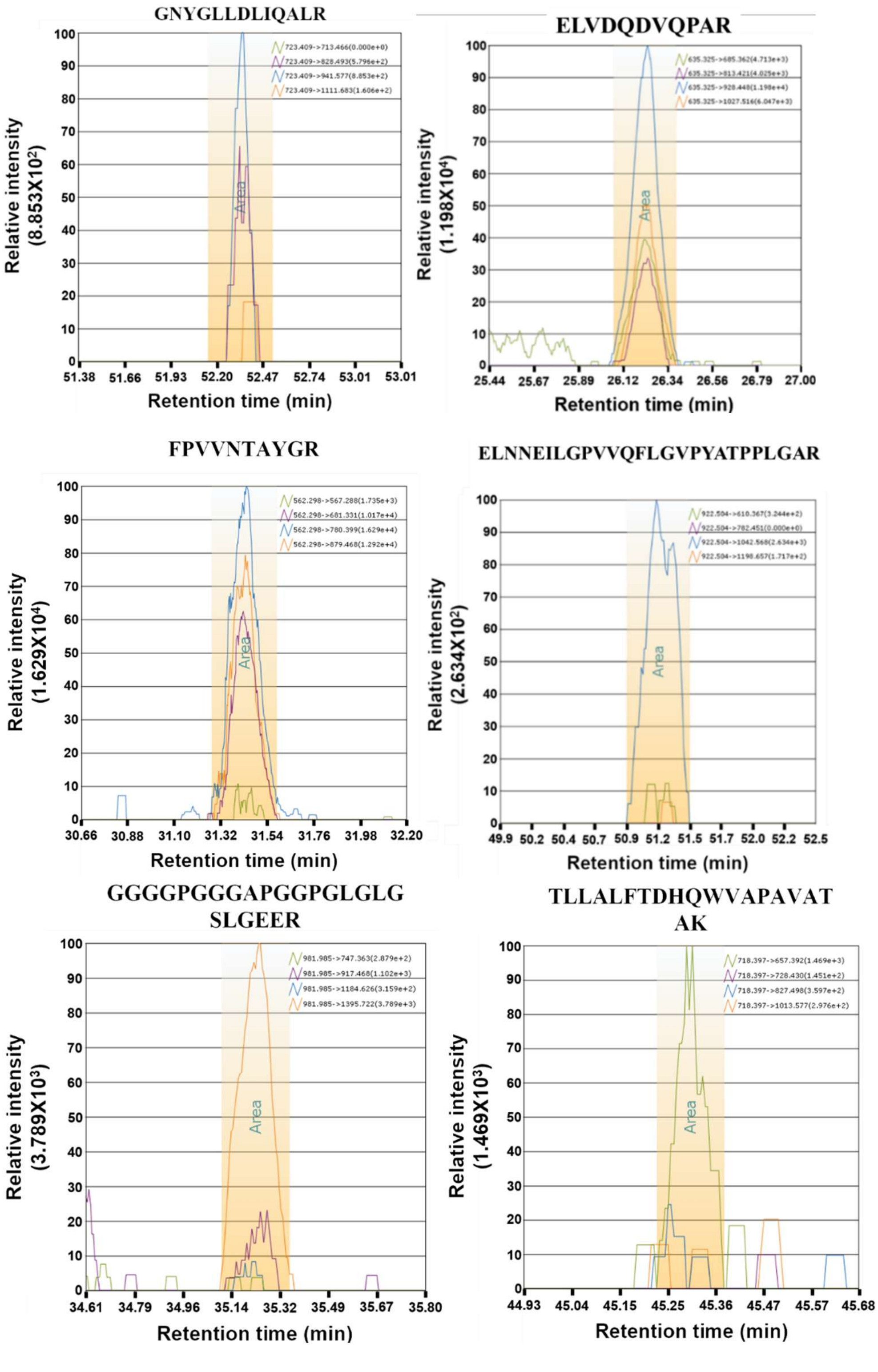

Supplementary Figure 1. NLGNs-enrichment assay chromatograms. Representative chromatogram of each detected peptide in plasma samples, showing endogenous peptide transitions. The x-axis shows elution time in minutes.

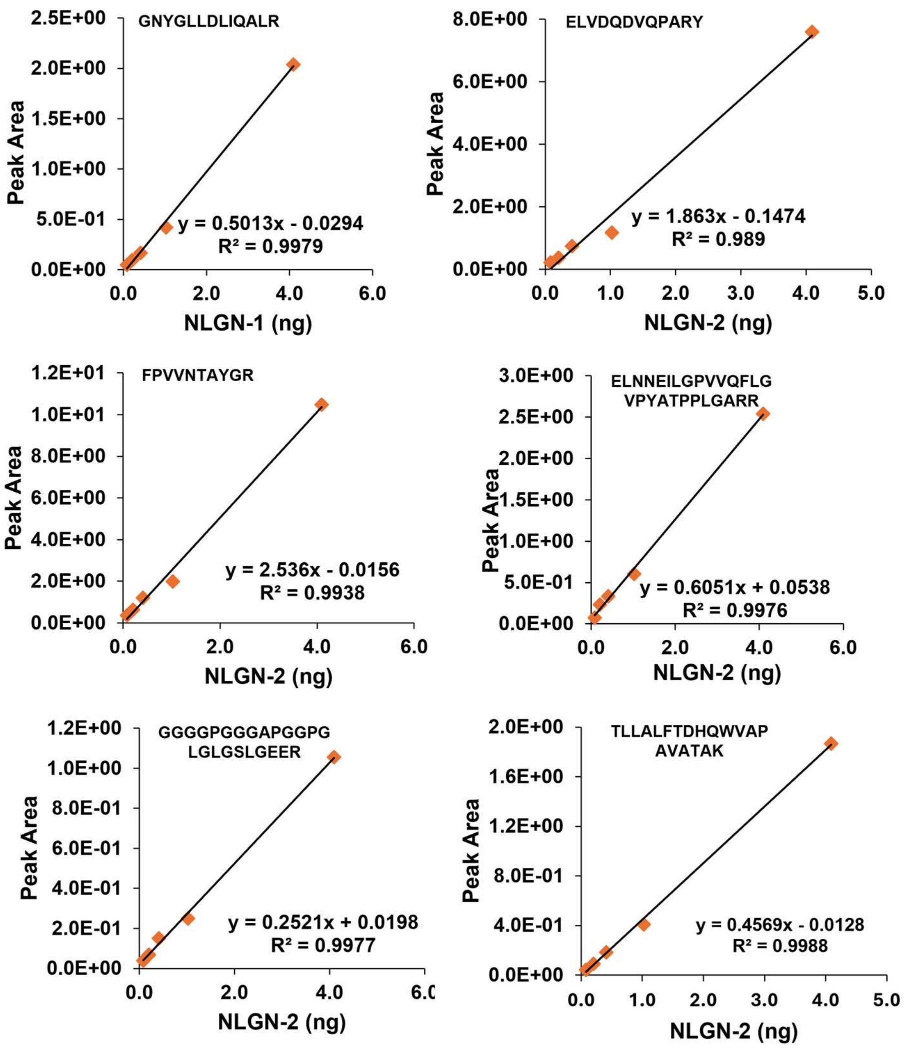

Supplementary Figure 2. Standard curves with recombinant NLGNs. Representative recombinant NLGNs standard curves for peptides over their linear range. The y-axis peak area represents the value under the curve for each endogenous value relative to NLGN1 heavy isotope-labeled peptide reference (GNYGLLDLIQALR) or NLGN2 heavy isotope-labeled peptide reference (FPVVNTAYGR).

NLGN1 (excitatory synapses)

NLGN1(267-279)

MALPRCTWPNYVWRAVMACLVHRGLGAPLTLCMLGCLLQAGHVLSQKLDDVDPLVATNFGKIRGIKKELNNEILGPVIQFLGVPYAAPPTGERRFQPPEPPSPWSDIRNA
TQFAPVCPQNIIDGRLPEVMLPVWFTNNLDVVSSYVQDQSEDCLYLNIYVPTEDVKRISKECARKPGKKICRKGGPLTKKQTDDLGDNDGAEDEDIRDSGGPKPVMVYIHG
GSYMEGTGNLYDGSVLASYGNVIVITVNYRLGVLGFLSTGDQAAKGNYGLLDLIQALRWTSENIGFFGGDPLRITVFGSGAGGSCVNLLTLSHYSEGNRWSNSTKGLFQRAI
AQSGTALSSWAVSFQPAKYARMLATKVGCNVSDTVELVECLQKKPYKELVDQDIQPARYHIAFGPVIDGDVIPDDPQILMEQGEFLNYDIMLGVNQGEGLKFVENIVDSD
DGISASDFDFAVSNFVDNLYGYPEGKDVLRETIKFMYTDWADRHNPETRRKTLLALFTDHQWVAPAVATADLHSNFGSPTYFYAFYHHCQTDQVPAWADAAHGDEVPY
VLGIPMIGPTELFPCNFSKNDVMLSAVVMTYWTNFAKTGDPNQPVPQDTKFIHTKPNRFEEVAWTRYSQKDQLYLHIGLKPRVKEHYRANKVNLWLELVPHLHNLNDIS
QYTSTTTKVPSTDITFRPTRKNSVPVTSAFPTAKQDDPKQQPSPFSVDQRDYSTELSVTIAVGASLLFLNILAFAALYYKKDKRRHDVHRRCSPQRTTTNDLTHAQEEEIMSLQ
MKHTDLDHECESIHPHEVVLRTACPPDYTLAMRRSPDDVPLMTPNTITMIPNTIPGIQPLHTFNTFTGGQNNTLPHPHPHPHSHSTTRV

NLGN2 (inhibitory synapses)

NLGN2(17-40) NLGN2(41-50) NLGN2(57-82)

MWLLALCLVGLAGAQRGGGGPGGGAPGGPGLGLGSLGEERFPVVNTAYGRVRGVRRELNNEILGPVVQFLGVPYATPPLGARRFQPPEAPASWPGVRNATTLPPACPQN
LHGALPAIMLPVWFTDNLEAAATYVQNQSEDCLYLNLYVPTEDGPLTKKRDEATLNPPDTDIRDPGKKPVMLFLHGGSYMEGTGNMFDGSVLAAYGNVIVATLNYRLGVLG
FLSTGDQAAKGNYGLLDQIQALRWLSENIAHFGGDPERITIFGSGAGASCVNLLILSHHSEGLFQKAIAQSGTAISSWSVNYQPLKYTRLLAAKVGCDREDSAEAVECLRRKPS

NLGN2(336-346)

RELVDQDVQPARYHIAFGPVVDGDVVPDDPEILMQQGEFLNYDMLIGVNQGEGLKFVEDSAESEDGVSASAFDFTVSNFVDNLYGYPEGKDVLRETIKFMYTDWADRDN

NLGN2(450-469)

GEMRRKTLLALFTDHQWVAPAVATAKLHADYQSPVYFYTFYHHCQAEGRPEWADAAHGDELPYVFGVPMVGATDLFPCNFSKNDVMLSAVVMTYWTNFAKTGDPNQP
VPQDTKFIHTKPNRFEEVVWSKFNSKEKQYLHIGLKPRVRDNYRANKVAFWLELVPHLHNLHTELFTTTTRLPPYATRWPPRPPAGAPGTRRPPPPATLPPEPEPEPGPRAYD
RFPGDSRDYSTELSVTVAVGASLLFLNILAFAALYYKRDRRQELRCRRLSPPGGSGSGVPGGGPLLPAAGRELPPEEELVSLQLKRGGGVGADPAEALRPACPPDYTLALRRAP
DDVPLLAPGALTLLPSGLGPPPPPPPPSLHPFGPFPPPPPTATSHNNTLPHPHSTTRV

Supplementary Figure 3. Position of the peptides measured in NLGN1 and NLGN2 amino acid sequence. The extracellular domain is in red.

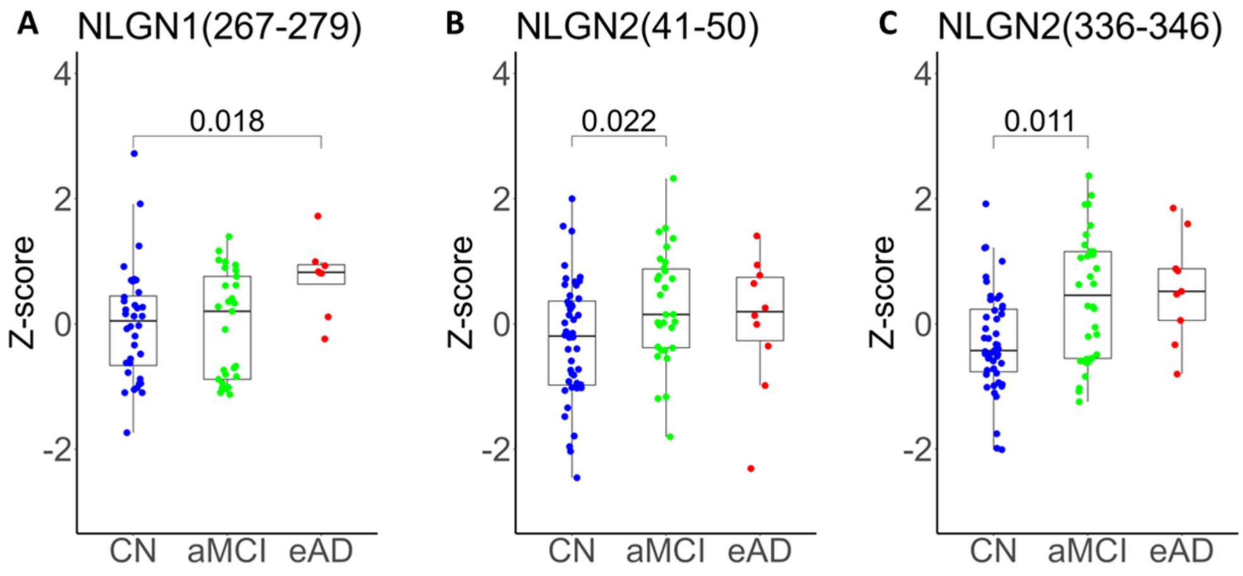

Supplementary Figure 4. Plasma levels of NLGN-associated peptides (A) NLGN1(267-279), (B) NLGN2(41-50), and (C) NLGN2(336-346) in cognitively normal (CN) individuals compared to individuals with amnestic mild cognitive impairment (aMCI) and early Alzheimer's disease (eAD). Z-score corresponds to the normalized value corrected for batch effect. Statistical differences were assessed using ANCOVA including age, sex and APOE genotype as covariates ((NLGN241-50) and NLGN2(336-346)) and the Kruskal-Wallis test (NLGN1(267-279)). P-values were adjusted for false discovery rate (FDR). CN: cognitively normal, aMCI: amnestic mild cognitive impairment, eAD: early Alzheimer's disease.

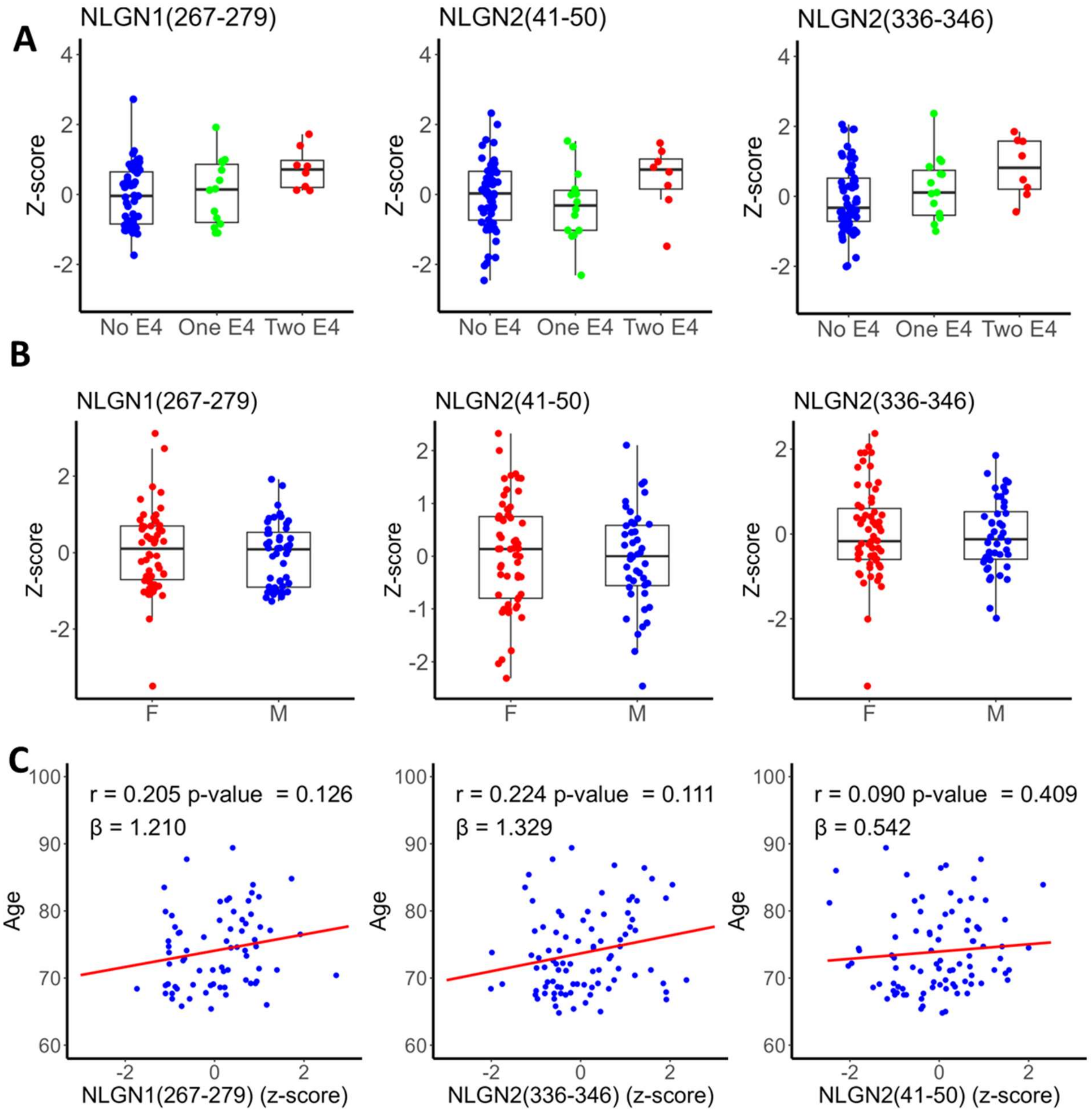

Supplementary Figure 5. Plasma concentration of NLGN peptides by APOE genotype, sex and age. (A) Plasma levels of NLGN-derived peptides according to APOE4 number of alleles **(B)** Comparison of NLGN peptide levels in blood by sex. Z-score corresponds to the normalized value corrected for batch effect. Statistical differences were assessed using ANCOVA, including age, and sex or APOE genotype as covariates. **(C)** Correlation between plasma concentration of NLGN peptides and age. Sex and APOE genotype were included as covariates. The partial Pearson correlation coefficient, the beta coefficient and significant levels are indicated in the top of each plot. Scale of NLGN fragments is the Z-score that corresponds to the normalized value corrected for batch effect. P-values were adjusted for FDR. E4 – APOE4 allele, F – female, M – male.

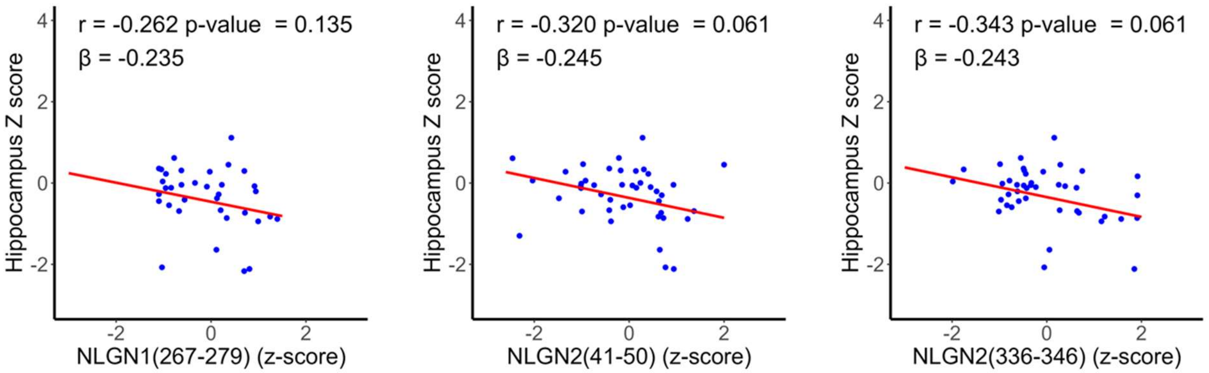

Supplementary Figure 6. Plasma concentration of NLGN-derived peptides in relation to the size of hippocampus. **(A-C)** Correlations between plasma concentration of NLGN-derived peptides, and the normalized size of hippocampus. Correlations were derived from linear models including age, sex and APOE genotype as covariates. Pearson partial correlation coefficients, significance levels, and beta coefficients are shown at the top of each plot. NLGN peptide levels are expressed as Z-scores, normalized and corrected for batch effects. P-values were adjusted for FDR.

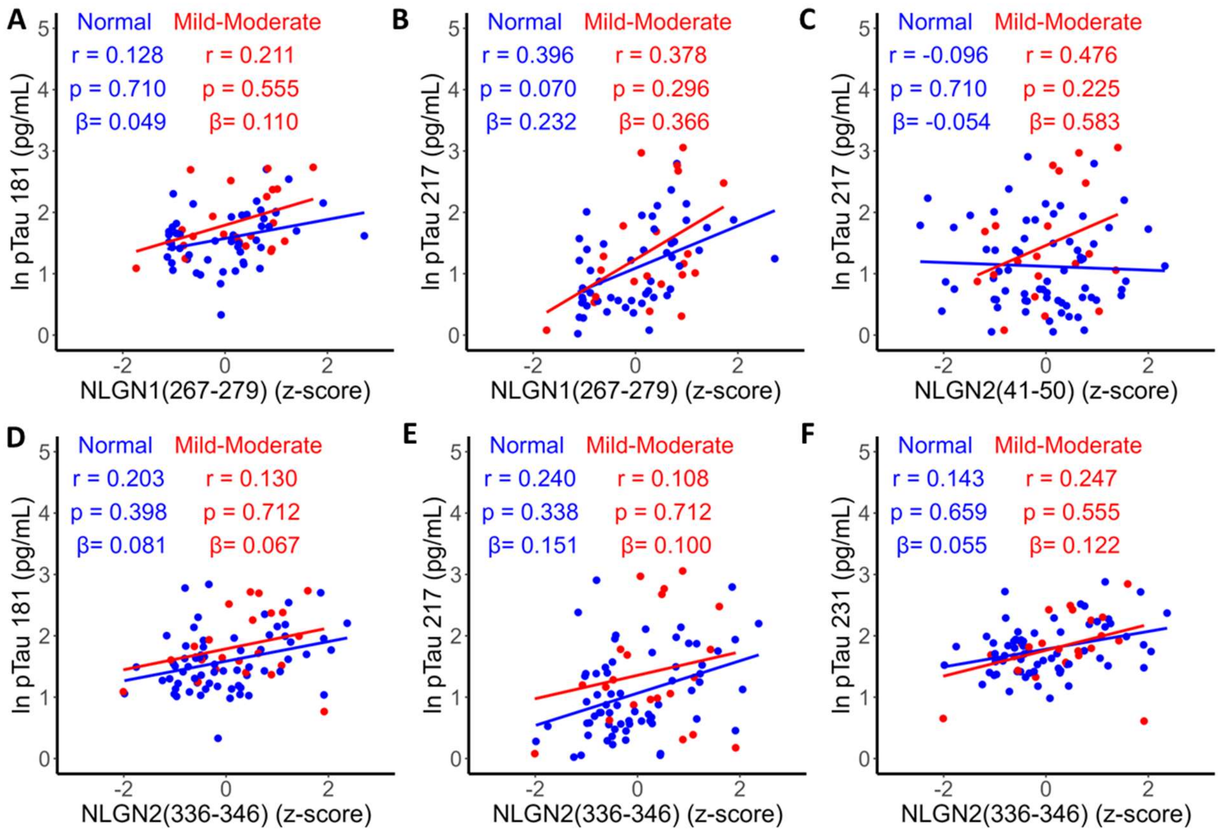

Supplementary Figure 7. Plasma levels of NLGN-associated peptides in relation to Mini-Mental State Examination (MMSE) score and pTau. (**A-F)** Correlations between plasma levels of NLGN-derived peptides and plasma levels of phosphorylated Tau 181, 217 and 231, stratified by cognitive severity based on MMSE score. Participants were grouped into two categories: normal (MMSE 30-25), mild-moderate (MMSE 24-10). Correlations were derived from linear models including age, sex and APOE genotype as covariates. Pearson partial correlation coefficients, significance levels, and beta coefficients are shown at the top of each plot. NLGN peptide levels are expressed as Z-scores, normalized and corrected for batch effects. P-values were adjusted for FDR.
