# Supplementary figures and images for: Differences in blood levels of neuroligin-derived peptides in a cohort for early detection of Alzheimer’s disease
Source: J Gerontol A Biol Sci Med Sci. 2026 Feb 3;81(4):glag009. doi: 10.1093/gerona/glag009 (PMC13049705; doi:10.1093/gerona/glag009)

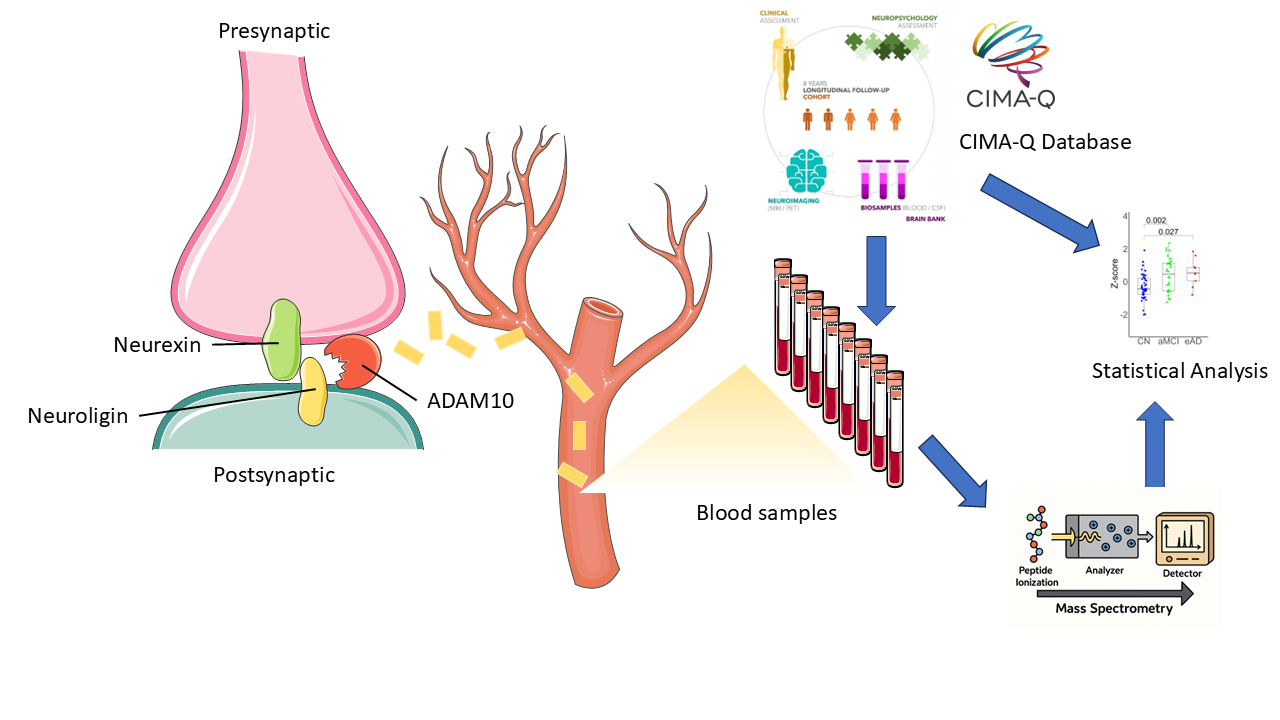

Supplement: glag009_Supplementary_Data [file glag009_supplementary_data.zip › Graphical abstract.png]
